# Next-Generation Vaccine Design for Porcine Enteric Coronaviruses: Aligning Antigenic Breadth, Mucosal Immunity, and Translational Evaluation

**DOI:** 10.3390/vaccines14060498

**Published:** 2026-06-02

**Authors:** Fanzhi Kong, Nannan Wu, Shuxuan Liang, Yufeng Yan

**Affiliations:** 1 College of Animal Science and Veterinary Medicine, Heilongjiang Bayi Agricultural University, No. 5 Xinfeng Road, Sartu District, Daqing 163319, China; wunannan0420@byau.edu.cn (N.W.); lsx17545595612@hotmail.com (S.L.); yanyufeng0314@byau.edu.cn (Y.Y.); 2 XL Future AgriTech, National University Science Park, Northeast Petroleum University, No. 38 Huoju Xinjie, Daqing High-Tech Zone, Daqing 163000, China

**Keywords:** porcine enteric coronavirus, PEDV, TGEV, PDCoV, SADS-CoV

## Abstract

Porcine enteric coronaviruses (PECs), including porcine epidemic diarrhea virus (PEDV), transmissible gastroenteritis virus (TGEV), porcine deltacoronavirus (PDCoV), and swine acute diarrhea syndrome coronavirus (SADS-CoV), remain major causes of neonatal diarrhea, dehydration, mortality, and economic loss in swine production. Despite substantial progress in vaccine development, durable field protection is still inconsistent. In this narrative review, this narrative review synthesizes current knowledge on PEC vaccine design from three connected perspectives: antigenic breadth, mucosal immunity, and translational evaluation. The economic and virological context of PEC vaccine development is first summarized, including the recurrent production burden of PECs, coronavirus genome organization, structural proteins, and the central role of the spike protein in receptor engagement, membrane fusion, and neutralizing antibody induction. Key issues are then discussed, including how spike diversity, conformational stability, epitope accessibility, glycan shielding, and antigen matching influence protective breadth; why intestinal secretory IgA, mucosal immune-cell trafficking, local memory responses, and lactogenic immunity should be prioritized as biologically relevant endpoints; and how delivery route, adjuvant selection, and platform design shape response quality. Current evidence on recombinant protein, viral-vectored, nanoparticle, virus-like particle, probiotic, plant-derived, and mRNA-based approaches is compared with attention to both promise and current evidentiary and translational limitations. The available literature suggests that future progress in PEC vaccinology is likely to depend less on platform novelty alone than on integrated vaccine designs that align antigen selection, mucosal delivery, maternal–neonatal protection, heterologous challenge, manufacturability, and field applicability.

## 1. Introduction

Porcine enteric coronaviruses (PECs) continue to cause substantial losses in the swine industry despite improvements in surveillance, biosecurity, and vaccination practice [1,2,3,4]. Porcine epidemic diarrhea virus (PEDV) and transmissible gastroenteritis virus (TGEV) remain the most extensively studied members of this group, whereas porcine deltacoronavirus (PDCoV) and swine acute diarrhea syndrome coronavirus (SADS-CoV) have further expanded the biological and control challenges by adding additional genetic and antigenic diversity [5,6]. The economic relevance of PEC vaccines should be considered in the broader context of major swine infectious diseases. Unlike African swine fever, which can generate acute national and international market shocks, PECs more commonly impose recurrent production-level losses through neonatal mortality, diarrhea, growth retardation, increased biosecurity costs, emergency vaccination, and impaired sow–piglet herd stability. The 2013–2014 PEDV epidemic in the United States caused marked supply-side disruption, while PRRS continues to impose large recurrent losses in commercial pig production [7,8,9]. Thus, the business case for PEC vaccines lies not only in preventing major outbreaks, but also in reducing repeated herd-level losses and improving predictable maternal–neonatal protection under routine production conditions.

In neonatal piglets, where morbidity and mortality are greatest, vaccine-mediated protection must be achieved in the setting of an immature immune system, rapid viral amplification in the intestinal epithelium, and the practical constraints of sow–piglet management. These features make PEC vaccination a demanding problem in applied vaccinology rather than a straightforward extension of conventional parenteral vaccine design. A recurrent weakness in the literature is the tendency to equate measurable serum neutralizing activity, or partial improvement after homologous challenge, with meaningful vaccine progress. For PECs, that threshold is insufficient. A useful vaccine must protect at the intestinal mucosa, maintain efficacy against epidemiologically relevant strains, and function under realistic maternal-immunity and production conditions. Taken together, the literature points to two interlocking bottlenecks: (1) Spike (S), the dominant target of neutralizing antibodies, is also the most variable and conformationally sensitive viral antigen, so vaccine-induced immunity can be narrowed by antigenic drift, glycan shielding, or presentation of non-native epitopes [10,11,12,13]; (2) Immunological: PEC infection is initiated at the intestinal surface, yet vaccine evaluation has often depended on systemic delivery and serum-based readouts that only partially capture mucosal protection [14,15,16]. Accordingly, the most credible path forward is not simply to adopt newer platforms, but to align antigen design, route, and efficacy assessment with the biological requirements of enteric protection.

A recurring limitation in the current literature is that antigen design, mucosal immunobiology, and translational evaluation are often discussed separately. In practice, these elements are interdependent. Spike-based immunogens must be selected and presented in a way that preserves relevant neutralizing epitopes; delivery routes and adjuvants must support intestinal or linked mucosal responses; and vaccine evaluation must include endpoints that reflect enteric protection, such as secretory IgA, fecal viral shedding, diarrhea severity, lactogenic immunity, and heterologous challenge. Accordingly, the most viable path for next-generation PEC vaccines is not simply to adopt newer platforms, but to align antigen design, route of immunization, and efficacy assessment with the biological requirements of enteric protection. This narrative review discusses the development of next-generation PEC vaccines by focusing on antigenic breadth, mucosal immunity, and translational evaluation. We first introduce the economic, virological, and structural basis of PEC vaccine design, then discuss spike antigen selection, route-dependent mucosal protection, emerging vaccine platforms, current evaluation gaps, and future priorities for field-relevant vaccine development.

## 2. Virological and Structural Basis of PEC Vaccine Design

PECs are enveloped, positive-sense, single-stranded RNA viruses. Their genomes typically contain a 5′ untranslated region, a large replicase region, genes encoding structural proteins, virus-specific accessory genes, and a 3′ untranslated region followed by a poly(A) tail [17]. The 5′ two-thirds of the genome encode the replicase polyproteins pp1a and pp1ab, which are proteolytically processed into nonstructural proteins that form the viral replication–transcription complex. These nonstructural proteins include enzymes and regulatory factors involved in RNA synthesis, proofreading, protease activity, membrane rearrangement, and modulation of host antiviral responses. Although these proteins are essential for viral replication and pathogenesis, they are generally less accessible to antibodies and therefore are not the primary targets of most neutralizing vaccine strategies [18]. The virion contains four major structural proteins: spike (S), envelope (E), membrane (M), and nucleocapsid (N). The N protein binds viral genomic RNA and contributes to nucleocapsid formation. The M protein is the most abundant envelope protein and plays a central role in virion assembly and morphogenesis. The E protein is a small envelope-associated protein involved in virion assembly, release, and virus–host interaction. In contrast, the S protein forms large surface projections on the virion and is directly responsible for receptor engagement and membrane fusion. Because it is exposed on the viral surface and contains major neutralizing epitopes, S is the dominant antigenic target for most PEC vaccine designs [19].

The S protein is a type I transmembrane glycoprotein that can be functionally divided into S1 and S2 regions. The S1 region is primarily involved in receptor binding and contains several major antigenic and highly variable regions, whereas the S2 region contains the fusion machinery, including the fusion peptide, heptad repeat regions, transmembrane domain, and cytoplasmic tail [20]. This organization is highly relevant to vaccine design. Antigens derived from S1 or receptor-binding regions may induce potent neutralizing antibodies but can be vulnerable to antigenic drift and strain restriction. In contrast, S2-related regions are generally more conserved but may be less immunodominant or less accessible [21]. Therefore, rational PEC vaccine design requires careful balancing of antigenic specificity, conformational fidelity, epitope accessibility, and potential breadth of protection. PEDV, TGEV, PDCoV, and SADS-CoV differ in genome organization, spike sequence, receptor usage, and antigenic relatedness. These differences limit the likelihood that a single unmodified spike antigen will provide broad protection across all PECs. At the same time, their shared enteric tropism and mucosal pathogenesis create common requirements for vaccine-induced protection, including intestinal secretory IgA, local immune memory, reduced fecal shedding, and maternal antibody transfer in sow-based immunization programs. These virological and structural considerations provide the foundation for the antigen-design issues discussed below.

## 3. Antigen Selection and Immunogen Design

**Figure 1:**
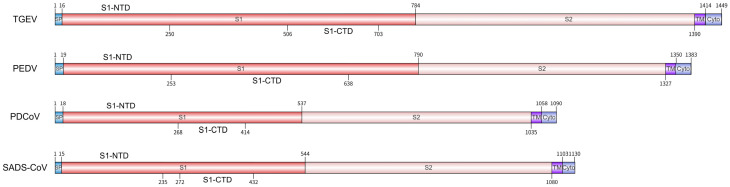
Comparative spike protein organization of major porcine enteric coronaviruses. The spike proteins of PEDV, TGEV, PDCoV, and SADS-CoV share a general S1–S2 organization but differ substantially in domain composition and sequence variability. S1 is mainly associated with receptor engagement and antigenic variation, whereas S2 contains fusion-related regions.

On the basis of the virological and structural features described above, the S protein remains the most important antigenic target for PEC vaccine development [11,13]. However, spike should not be treated as a single interchangeable antigen across PEDV, TGEV, PDCoV, and SADS-CoV. Protective performance depends not only on including spike-derived sequences, but also on selecting appropriate domains, preserving prefusion or native-like conformational epitopes, maintaining relevant glycan context, and matching the immunogen to contemporary circulating strains. The uneven distribution of sequence variability across spike is particularly important. S1 contains receptor-binding and antigenically variable regions and is therefore often associated with strain-specific neutralization (Figure 1). S2 contains the fusion machinery and is relatively more conserved, but conserved regions may be less accessible or less immunodominant. In the prefusion spike trimer, several S2 elements are located in membrane-proximal or conformationally constrained regions, and some cryptic S2 epitopes become accessible only during spike breathing or receptor-triggered conformational transitions, which may limit steady-state antibody access [22]. Moreover, potential N-linked glycosylation sequons may shape epitope exposure by masking nearby peptide surfaces and contributing to local antigenic architecture [23]. Thus, the key issue is not whether spike should be used, but how spike-based immunogens should be selected, stabilized, displayed, and benchmarked against contemporary sequence diversity, glycan shielding, structural accessibility, and heterologous challenge.

Structural studies have substantially strengthened the field by resolving PEDV, PDCoV, and SADS-CoV spike architecture and clarifying which surfaces are accessible or shielded from antibody recognition [11,12,24]. Nevertheless, structural insight does not by itself generate better vaccines. Candidate immunogens are still frequently advanced without a clear demonstration that they preserve the most relevant conformational epitopes or reduce immunofocusing toward strain-restricted regions. More focused immunogens, including receptor-binding domain (RBD), S1 C-terminal domain, or selected S2-based constructs, offer potential advantages in manufacturability and epitope targeting [25,26]. Yet a narrower antigen is not automatically a better antigen. It may improve precision while sacrificing epitope completeness, especially if protection depends on cooperative recognition of multiple spike surfaces. The most promising direction is likely a structurally informed middle ground: antigens that retain the conformational determinants most relevant to cross-neutralization while minimizing distracting or unstable regions. The key question is no longer whether spike should remain central, but how rigorously spike-based immunogens are being optimized and benchmarked against structural evidence and contemporary sequence diversity.

## 4. Mucosal Immunity and Route of Immunization

**Figure 2:**
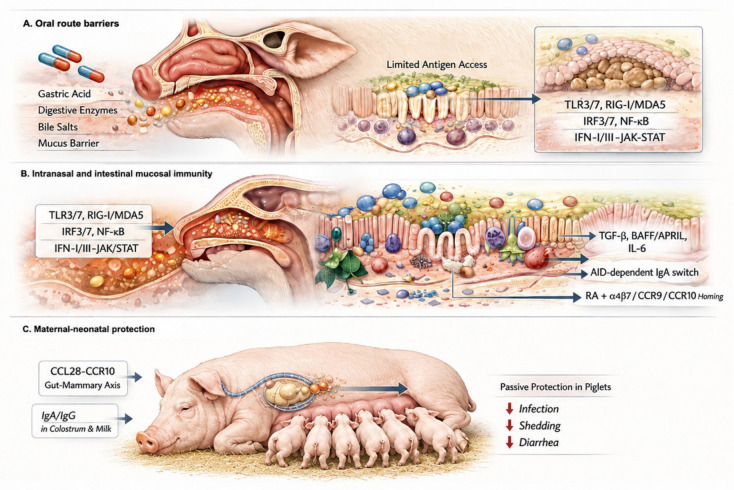
Route-dependent mucosal and maternal protection against PECs. (**A**) Oral and (**B**) intranasal immunization may induce mucosal immune responses through different anatomical and immunological routes. Effective protection against PECs is expected to depend on intestinal secretory IgA, mucosal immune-cell trafficking, local memory responses, and (**C**) lactogenic antibody transfer from immunized sows to neonatal piglets. The schematic figures were initially generated or refined with the assistance of AI-based image tools and were subsequently modified and verified by the authors. The authors take full responsibility for the scientific accuracy of all figure content.

For enteric coronaviruses, route of immunization is a primary design variable because the intestine is both the portal of entry and the principal site of viral amplification. This principle is well supported in PEDV and related enteric coronavirus research, yet it is still not consistently reflected in PEC vaccine development [27,28]. Studies continue to privilege serum IgG and peripheral neutralization titers, even though the most relevant effectors of protection are secretory IgA, mucosal plasmablast trafficking, tissue-resident memory responses, and, in production settings, lactogenic immunity transferred from immunized sows [29,30,31]. Accordingly, a vaccine candidate that performs well systemically but fails to establish strong mucosal immunity should be regarded as only partially successful rather than broadly protective. Mucosal vaccination remains intrinsically challenging because orally delivered antigens must survive gastric acidity, digestive enzymes, bile salts, and mucus barriers, while also avoiding tolerogenic signaling in the intestinal environment before reaching inductive sites such as Peyer’s patches [32,33]. After oral delivery, antigens must retain sufficient integrity and dose while passing through gastric acid, digestive enzymes, bile salts, and the mucus layer before reaching intestinal inductive sites such as Peyer’s patches (Figure 2A). Successful mucosal priming is therefore not determined by antigen sequence alone, but also by whether the delivery system protects the antigen and directs it to the appropriate epithelial and immune-cell compartments. At these sites, engagement of mucosal innate sensing pathways, including TLR3/7- and RIG-I/MDA5-associated recognition, can activate IRF3/7, NF-κB, and downstream type I and type III interferon responses. These early signals may help establish an antiviral mucosal environment and shape the quality of adaptive immunity [34,35]. In parallel, induction of durable intestinal antibody-mediated protection requires efficient IgA programming, likely involving transforming growth factor-β (TGF-β-), B-cell activating factor/a proliferation-inducing ligand (BAFF/APRIL-), and interleukin-6 (IL-6)-associated signals that support acquired immunodeficiency syndrome (AID)-dependent IgA class switching, followed by pIgR-mediated epithelial transcytosis of dimeric IgA and release of secretory IgA at the mucosal surface [36,37,38,39]. For this reason, many oral formulations fail not because the underlying antigen is weak, but because the delivery system does not adequately preserve dose, timing, biodistribution, and access to the signaling microenvironments required for intestinal immune induction.

Intranasal immunization deserves careful attention because it may activate the common mucosal immune system while avoiding several barriers faced by oral vaccines [28,40]. However, although nasal and intestinal mucosal compartments are linked through the common mucosal immune system, they are not functionally interchangeable; accordingly, intranasal immunogenicity should not be assumed to translate automatically into robust intestinal protection in neonatal piglets, particularly in the absence of challenge-based and field-relevant validation [28,41]. Mechanistically, any protective benefit of intranasal vaccination for PECs would still need to converge on intestinal effector pathways, including mucosal homing and local immune positioning (Figure 2B). Although intranasal vaccination may engage the common mucosal immune system, nasal immune activation should not be assumed to be equivalent to intestinal protection. For PECs, protective intranasal responses would still need to generate effector cells capable of trafficking to intestinal sites. Retinoic acid-imprinted α4β7-, CCR9-, and CCR10-associated homing programs are therefore important because they help direct activated plasmablasts and lymphocytes toward intestinal effector tissues, where they can support local sIgA production and memory responses [42,43]. Direct comparison of oral, intranasal, and prime–boost combinations using matched antigens and standardized endpoints would therefore be more informative than the current literature, which often evaluates these routes in isolation. Live-vectored approaches remain attractive because they can deliver antigen in an immunologically active context and may better support local priming [44,45]. Nevertheless, they also raise familiar concerns regarding pre-existing vector immunity, attenuation stability, dose control, and regulatory acceptability. In sow vaccination programs, an additional practical question is whether a given mucosal strategy can reliably enhance colostral and milk-derived protection for piglets [29,46,47]. In sow vaccination programs, mucosal immunity must also be evaluated as part of a maternal–neonatal protection system. The gut–mammary axis is particularly relevant because antigen-activated IgA-secreting cells induced at mucosal sites may traffic to the mammary gland. CCL28–CCR10-associated recruitment may contribute to the accumulation of IgA-producing cells in mammary tissue, thereby enhancing the transfer of IgA and IgG through colostrum and milk. These lactogenic antibodies are important for neonatal piglets because they can provide passive protection during the period when the piglet immune system is still immature. Therefore, sow vaccine evaluation should include colostrum and milk IgA/IgG, milk neutralizing activity, duration of maternal protection, piglet diarrhea, fecal shedding, and survival (Figure 2C).

## 5. Integrated Vaccine Platforms

One of the most encouraging developments in recent PEC vaccine research is the growing convergence of antigen engineering, delivery technology, and formulation science. Rather than viewing these as separate layers, stronger studies increasingly suggest that vaccine performance emerges from their interaction [48,49,50]. This shift is conceptually important because it moves the field away from asking which platform is intrinsically “best” and toward asking which integrated design is most fit for purpose. mRNA platforms illustrate both the promise and the constraints of next-generation PEC vaccine development. Their rapid redesign cycle, capacity for in situ antigen expression, and compatibility with iterative spike updating make them attractive for drift-prone enteric coronaviruses [51,52]. However, platform flexibility does not eliminate the need for rigorous antigen validation, dose optimization, cold-chain realism, manufacturing feasibility, and cost-sensitive deployment. In veterinary settings, novelty is valuable only if it remains practical under commercial conditions. For large-scale swine production, this also requires consideration of batch-to-batch consistency, dose volume, administration labor, compatibility with sow and piglet vaccination schedules, regulatory approval pathways, and the ability to maintain potency during storage and farm-level distribution.

Nanoparticles and virus-like particles (VLPs) offer distinct advantages, particularly multivalent display, efficient B-cell engagement, and modular compatibility with mucosal delivery strategies [53]. These features are biologically appealing, but increased immunogenicity does not necessarily translate into broader protection or superior field performance. Strong preclinical readouts may reflect platform-enhanced magnitude rather than qualitatively better immunity. Head-to-head comparisons with matched antigen payloads therefore remain essential. Viral-vectored systems also remain attractive because they can deliver antigen in an immunologically active context, engage innate sensing pathways, and may better support local priming, particularly when combined with mucosal delivery strategies [18,54]. However, these potential advantages must be weighed against pre-existing vector immunity, attenuation stability, dose control, biosafety considerations, and regulatory constraints. Recombinant protein subunits remain an important benchmark because they are comparatively tractable to manufacture, characterize, and scale [55,56]. Although they often show weaker mucosal induction and greater dependence on formulation, these limitations do not negate their translational strengths. For PEC vaccines, simpler platforms may ultimately prove more competitive if they combine acceptable antigen fidelity, manufacturability, stability, and compatibility with routine herd vaccination.

**Figure 3:**
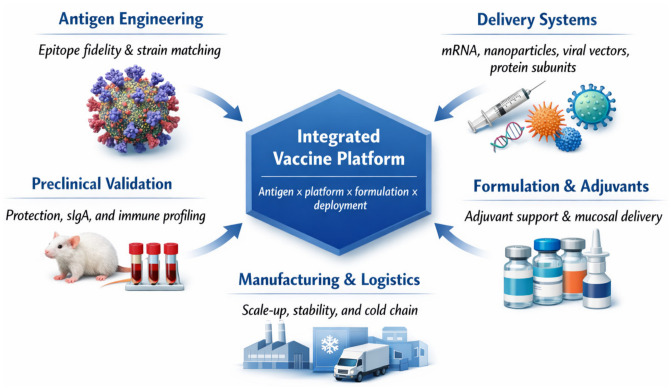
Integrated framework for next-generation PEC vaccine design. Field-relevant vaccine performance depends on coordinated antigen engineering, delivery route, formulation, preclinical validation, and manufacturing feasibility. The goal is not platform novelty alone, but an integrated design capable of inducing mucosal protection while remaining practical for veterinary deployment. The schematic figures were initially generated or refined with the assistance of AI-based image tools and were subsequently modified and verified by the authors. The authors take full responsibility for the scientific accuracy of all figure content.

An integrated platform should therefore be evaluated across several linked dimensions rather than by platform label alone. Antigen engineering determines whether the immunogen preserves relevant epitopes and matches circulating strains. The delivery system shapes antigen biodistribution, uptake, and mucosal access. Formulation and adjuvant selection influence innate activation, response magnitude, and durability. Preclinical validation should test not only antibody titers, but also protection, intestinal sIgA, fecal viral shedding, and clinical outcomes. Finally, manufacturing and logistics determine whether a candidate can be produced reproducibly, stored stably, transported through realistic cold-chain conditions if required, and administered efficiently in large herds. These dimensions together determine whether a vaccine is merely immunogenic in an experimental setting or genuinely deployable in commercial swine production (Figure 3).

## 6. Critical Gaps in Evaluation and Translation

Evidence strength should be interpreted cautiously across the PEC vaccine literature. Some conclusions are supported by direct challenge studies in pigs, including reductions in diarrhea, fecal viral shedding, intestinal lesions, or piglet mortality. Other conclusions, particularly those involving newer platforms such as mRNA vaccines, nanoparticles, VLPs, recombinant probiotics, and plant-derived vaccines, are often based on preclinical models, limited challenge settings, or platform-specific proof-of-concept studies. Therefore, conceptual promise should not be interpreted as equivalent to field-ready efficacy. Differences in antigen design, delivery route, adjuvant formulation, animal model, challenge strain, sampling time, and immune endpoints make direct comparison among studies difficult. In this review, we distinguish between evidence-supported findings and future-oriented perspectives, and we emphasize the need for standardized comparative studies before any platform can be considered superior for PEC control. First, correlates of protection remain incompletely defined. Serum neutralizing titers are informative, but they cannot be assumed to represent intestinal secretory IgA, mucosal plasmablast recruitment and gut homing, local tissue-associated immune memory, or lactogenic transfer to piglets [42,43,57]. Studies lacking mucosal endpoints therefore provide only an incomplete basis for ranking vaccine candidates, especially when conclusions about protection are strong. In practical vaccine evaluation, protection should therefore be assessed using a combination of virological, clinical, pathological, maternal, and production-related endpoints. Virological endpoints may include fecal viral RNA shedding, duration of shedding, and infectious virus recovery when feasible. Clinical and pathological endpoints should include diarrhea severity, dehydration, survival, body-weight gain, intestinal lesion scoring, villus atrophy, villus-to-crypt ratio, and intestinal viral antigen distribution. For sow-centered vaccination, colostrum and milk IgA/IgG, milk neutralizing activity, duration of lactogenic protection, piglet diarrhea, fecal shedding, and survival are particularly relevant. Field-oriented studies should also consider production-related outcomes such as pre-weaning mortality, growth uniformity, medication use, and herd-level stability. Second, antigenic breadth remains insufficiently addressed. Homologous challenge is still overused relative to the diversity of viruses now encountered in the field. Although this approach can demonstrate internal consistency within an experimental system, it may overestimate likely performance under commercial conditions, particularly when heterologous cross-protection is limited or inconsistent [58,59]. For PEC vaccines, heterologous challenge panels, representative contemporary isolates, and explicit discussion of antigenic distance should be considered important features of preclinical evaluation rather than optional additions. Third, translational realism is still underemphasized. Veterinary vaccines must be affordable, stable, manufacturable at scale, and compatible with routine sow and piglet management, yet these issues are often treated only briefly. A platform that performs well experimentally but requires impractical formulation, complex cold-chain support, or unrealistic dosing schedules may still have limited field value. Stronger PEC studies should therefore treat manufacturability and deployment as scientific constraints rather than post hoc considerations. Overall, the available literature suggests that a major remaining gap is not simply the absence of vaccine concepts, but the lack of sufficiently rigorous and standardized frameworks to determine which candidates are protective, broadly relevant, and field deployable.

## 7. Priorities for Next-Generation PEC Vaccine Development

In the near term, a realistic strategy may be to refine existing vaccine concepts with greater biological precision rather than to replace them entirely. For PEDV and PDCoV, this means updating spike immunogens using contemporary sequence surveillance, structural information, and antigenic mapping; pairing these antigens with delivery systems that support intestinal or linked mucosal immunity; and evaluating success using endpoints relevant to both sow and piglet protection. Better alignment between immunogen choice and mucosal biology is likely to generate more value than platform diversification alone. This emphasis on sow-centered mucosal immunization is supported by work on lactogenic immunity, gestational timing, and host-factor regulation, which collectively indicate that milk IgA-linked protection is a mechanistic endpoint rather than a secondary readout [28,30,32,33].

In the medium term, the field would benefit more from rigorous comparative studies than from additional isolated proof-of-concept reports. mRNA, VLP, nanoparticle, vectored, and subunit platforms should be compared using matched or closely comparable antigens, harmonized dosing logic, shared immunological endpoints, and standardized challenge designs. Without such benchmarking, claims of platform superiority will remain difficult to interpret because differences in outcome may reflect antigen choice, delivery route, or adjuvant context rather than platform-specific advantages. This need is reinforced by both cautionary and encouraging findings: spike subunit immunization has, in some settings, exacerbated disease after challenge [40], whereas bivalent PEDV subunit vaccines, PEDV mRNA vaccines, recombinant probiotic approaches, and plant-derived maternal vaccines suggest that broader or maternally transferred protection is achievable when antigen design and delivery are aligned with the intended biological objective [58,60,61,62,63].

In the longer term, a broadly protective or modular pan-PEC strategy remains a rational but ambitious goal. A single universal vaccine may be difficult to achieve because PEDV, TGEV, PDCoV, and SADS-CoV differ substantially in genetic background, epidemiology, and likely correlates of protection [2,5]. However, a modular framework combining updated strain-matched antigens with shared mucosal delivery principles may be more feasible than a fully universal construct [64]. Progress toward this goal will depend on disciplined cross-strain evaluation rather than broad claims based primarily on design rationale. To avoid an overly descriptive comparison, Table 1 provides a concise summary of representative next-generation PEC vaccine strategies, focusing only on their main potential value, principal limitation, and representative supporting references.

## 8. Conclusions and Future Perspective

PEC vaccine development has reached a point at which further reformulation alone is unlikely to deliver consistent field benefit without clearer biological alignment. The current literature suggests that durable protection will depend on the convergence of antigenic relevance, mucosal effectiveness, and translational feasibility, rather than improvement in any one domain alone. Although mRNA, VLP, nanoparticle, and viral-vectored platforms remain promising, conceptual innovation should not be equated with practical deployability. Future progress is likely to require stricter benchmarking including contemporary antigen matching, route-appropriate immune endpoints, heterologous challenge, and realistic consideration of maternal immunity, manufacturability, scale-up, and field use. Overall, a key priority for next-generation PEC vaccinology is not simply to expand the range of candidate platforms, but to identify integrated vaccine designs that can provide biologically relevant, field-deployable, and commercially realistic protection. The main strength of this review is that it integrates antigenic breadth, mucosal immunobiology, maternal–neonatal protection, route-dependent immunization, and translational evaluation into a unified framework for PEC vaccine development. This perspective may help clarify why platform novelty alone is insufficient and why vaccine candidates should be evaluated according to mucosal relevance, heterologous protection, manufacturability, and field deployability. However, several limitations should also be acknowledged. This article is a narrative and translational synthesis rather than a systematic review or meta-analysis. PRISMA 2020 was used only as a general reference to improve transparency in describing the literature search process; no protocol registration, PRISMA-compliant systematic screening, formal risk-of-bias assessment, quantitative evidence grading, or meta-analysis was performed. Therefore, this review does not provide a formal quantitative ranking of vaccine platforms. In addition, the available evidence remains uneven across different PECs and vaccine technologies, and many next-generation candidates are still supported mainly by preclinical or limited challenge studies. More rigorous head-to-head comparisons using matched antigens, standardized mucosal endpoints, heterologous challenge, and field-relevant production outcomes will be needed before any platform can be considered clearly superior for commercial PEC control.

## Figures and Tables

**Table 1:** Concise comparison of representative next-generation PEC vaccine strategies.

Platform	Main Potential Value	Principal Limitation	References
Updated spike subunit vaccines	Practical antigen updating and relatively tractable manufacturing	Limited mucosal induction and possible strain restriction	[25,58]
mRNA vaccines	Rapid redesign and flexible in situ antigen expression	Cost, cold-chain requirement, dose optimization, and large-herd application logistics	[51,52,62]
Nanoparticle/VLP vaccines	Multivalent antigen display and enhanced B-cell engagement	Manufacturing complexity and insufficient validation of protection breadth	[26,48,53]
Viral-vectored vaccines	Active antigen delivery and potential support for local priming	Vector immunity, attenuation stability, biosafety, and regulatory constraints	[18,44,45,54]
Recombinant probiotic approaches and plant-derived approaches	Potential relevance for gut-directed or maternal vaccination	Variable potency, standardization, production consistency, and field validation	[50,60,61,63]
Mucosal prime–boost or modular strategies	Integration of antigen updating with route-adapted mucosal delivery	Complex schedules, benchmarking difficulty, and implementation challenges	[18,28,40,58,64]

## Data Availability

No new data were created or analyzed in this study.
